# Effect of initiation-inhibition and handedness on the patterns of the P50 event-related potential component: a low resolution electromagnetic tomography study

**DOI:** 10.1186/1744-9081-5-51

**Published:** 2009-12-24

**Authors:** Ion N Beratis, Andreas Rabavilas, Eleni D Nanou, Chrissanthi Hountala, Argiro E Maganioti, Christos N Capsalis, George N Papadimitriou, Charalabos Papageorgiou

**Affiliations:** 1 Eginition University Hospital, 1st Department of Psychiatry, Medical School, National University of Athens, 74 Vas. Sophias ave., 115 28 Athens, Greece; 2 University Mental Health Research Institute (UMHRI), 2 Soranou of Efesiou str., 156 01 Papagou, Athens, Greece; 3 Department of Electrical Engineering, Division of Information Transmission Systems and Material Technology, National Technical University of Athens, 9 Iroon Polytechneiou str., 157 80 Zografou Campus, Athens, Greece

## Abstract

**Background:**

Recent research recognizes the association between handedness, linguistic processes and cerebral networks subserving executive functioning, but the nature of this association remains unclear. Since the P50 event related potential (ERP) is considered to reflect thalamocortical processes in association with working memory (WM) operation the present study focuses on P50 patterns elicited during the performance of a linguistic related executive functioning test in right- and left-handers.

**Methods:**

In 64 young adults with a high educational level (33 left-handed) the P50 event-related potential was recorded while performing the initiation and inhibition condition of a modified version of the Hayling Sentence Completion test adjusted to induce WM. The manual preference of the participants was evaluated with the use of the Edinburgh Handedness Inventory (EHI).

**Results:**

P50 showed greater amplitudes in left- than in right-handers, mainly in frontal leads, in the initiation condition. Reduced amplitudes in inhibition compared to initiation condition were observed in left-handers. Low Resolution Electromagnetic Tomography (LORETA) analysis showed lower frontal lobe activation in the inhibition than in the initiation condition in both right- and left-handers. Also, LORETA yielded that right-handers exhibited greater activation in the inhibition condition than left-handers. Additionally, LORETA showed assymetrical hemispheric activation patterns in right-handers, in contrast to symmetrical patterns observed in left-handers. Higher P50 amplitudes were recorded in right-hemisphere of right-handers in the initiation condition.

**Conclusion:**

Brain activation, especially the one closely related to thalamocortical function, elicited during WM operation involving initiation and inhibition processes appears to be related to handedness.

## Background

The study of handedness is of interest because of its association with the lateralization of hemispheric function and the presence of data that indicate functional differences between left- and right-handers [1-3]. Although there is not complete agreement between the degree of right- and left-sided dominance of motor functions and lateralization of cognitive functions, a relationship between hemispheric asymmetry for components of cognition and handedness is evident.

Despite the fact that event related potential (ERP) research is very useful in furthering our knowledge of the brain's response to external stimuli, only a limited number of ERP neuropsychological studies have explored the association of handedness with electrophysiological activity; of those that have, conflicting results have been reported. Early ERP studies failed to find differences between right- and left-handers [4,5]. However, in a more recent report Coulson and Lovett [6] found differences between right- and left-handers in an ERP study on joke comprehension; left-handers manifested a late positivity (500-900 ms post-stimulus onset) that was larger and more broadly distributed than in right-handers, and in contrast to right-handers there was absence of asymmetry on the slow sustained negativity. Also, Nowicka et al. [7] by using visually presented words found differences in the ERP recordings between right- and left-handed women, observation which indicates that the encoding of new and repeated verbal information is differently lateralized in reference to handedness.

P50 is an early (30-80 ms) module of the ERP spectrum, and psychophysiological research has associated this ERP component with the gamma-band response (GBR) (20-50 Hz) [8,9]. In addition, because GBR is thought to originate from synchronized cortical networks engaged in working memory (WM) operations [10], and P50 is considered to reflect the gamma-band response, P50 is related to WM [11,12]. These studies reported alterations in P50 ERP component during a WM test, in association with memory performance [11], and the effect of mobile phone electromagnetic field [12]. Moreover, reports associate P50, in addition to GBR, and with low-frequency response (0-20 Hz) [13,14], and for this reason P50 is linked to new information encoding, detection of salient changes in sensory stimuli, and selective attention process as well [15].

Selective attention, WM, and inhibition, are higher-level cognitive functions, considered to comprise executive control, that are involved in the control and regulation of lower-level cognitive processing, in order to orchestrate cognitive performance [16,17]. These higher-level functions of cognition are crucial for skills required in everyday life, such as visuospatial accuracy and speed, inhibiting irrelevant stimuli, focusing on one single procedure, alternating attention between two simultaneous goals. Because of the importance of executive control in organizing, planning and monitoring behavior, there is a wide spectrum of neuropsychological tests used to measure and examine different aspects of this supervisory system of cognition. The Hayling Sentence Completion test is a relatively new measure of executive control designed to evaluate, with the use of speech stimuli, the ability to suppress a prepotent mandatory response that comes to mind [18,19].

The thalamus is found to be engaged in the processing of high-level linguistic features [20,21]. More specifically, thalamic structures are involved in the processing of syntactic and semantic aspects of auditory presented sentences constituting, in conjunction with cortical regions, language thalamocortical networks [20]. Also, the thalamus is associated with the P50 ERP, as this ERP component is thought to echo the synchronized response of the thalamocortical system. This is supported by recent research based on depth simulation and ERP recording suggesting a functional connectivity between the P50-like waveform and thalamocortical operation [22]. In light of the above considerations, and the reported functional differences in thalamic activity associated with handedness during a language task [3], we hypothesize that electrophysiological brain activity, as reflected by the P50 component, could be of value in identifying possible differences between the conditions of the Hayling Sentence Completion test adjusted to induce WM operation as well as between right- and left-handers. Contemporary neuropsychological views define WM as the capacity to keep information 'on-line' as necessary for an ongoing task [23,24]. Accordingly, WM is not for 'memorizing' per se; it is rather in the service of complex cognitive activities, such as reasoning, monitoring, problem solving, decision making, planning, and searching/shifting the initiation or inhibition response [25,26], thus comprising (among others) a central executive system. Therefore, the present study is designed to determine in a rather homogenous sample of young adults of high educational level whether or not: (a) there are different patterns of electrophysiological activity as reflected by P50 for each condition of the Hayling Sentence Completion test adjusted to induce WM operation, (b) there are different patterns of electrophysiological activity as recorded by P50 between right- and left-handers, (c) there are different patterns of electrophysiological activity between the left- and right-hemisphere in right- and left-handers, and (d) there is an interaction of any existing differences between handedness and the different conditions of the Hayling Sentence Completion test adjusted to induce WM.

## Methods

### Participants

After an initial gross screening for handedness, as described below, 71 individuals were asked to participate in the study, and all of them accepted. Additional criteria for the admission of the participants in the initial group of the study were to be physically healthy, without a history of neurologic or psychiatric disorder, or reading disabilities. Also, they had no reported history of illicit substance abuse or alcohol dependency. Those who failed to reach these criteria were excluded from the initial sample. The participants had university education or were university students, and their native language was Greek. For the selection from the initial group of those who were right-handers (100 to 50) and left-handers (-100 to 0), the manual preference of the participants was measured by the Edinburgh Handedness Inventory (EHI) [27], as described below. Of the initial group of subjects considered as possible participants in the study, 2 were excluded as ambidextrous. In addition, 5 individuals were excluded because of intense noise in the electroencephalogram (EEG) recording. Thus, a total of 64 individuals were analyzed in the study, 31(17 males) right-handed and 33 (17 males) left-handed. The mean age of the right-handers was 26.4 ± 3.1 (range, 22 to 34) and of the left-handers 25.3 ± 4.1 (range, 20 to 34) years.

The study was approved by the Ethics Committee of the Eginition University Hospital and informed consent was obtained from the subjects studied.

### Hayling Sentence Completion test

The modified version of the Hayling Sentence Completion test used in the present study is made up from two different conditions, response initiation, and response inhibition. In the response initiation condition, participants were instructed to complete auditory-presented sentences with a word clearly suggested by the context. Following are two indicative examples of the sentences presented and of the responses provided by the participants: "Captain Nicholas wanted to stay with the sinking ...", response "ship"; "Most cats see very well at ...", response "night". In the response inhibition condition, participants were instructed to produce a word that made no sense in the context of an auditory-presented sentence from which the last word was missing. Following are two indicative examples of the sentences presented and of the responses provided by the participants: "Water and sun help plants to ...", response "read "; "Don't believe in everything you ...", response "swim". The sentences were presented through earphones to the participants, the initiation and inhibition condition were examined separately, and the administration order of the two conditions was counterbalanced. The duration of the sentences was from 3-5 sec. After the presentation of each sentence, there was a 500 ms EEG recording period, then a warning stimulus (100 ms duration, 65 dB, 500 Hz) was given, followed by an interval of 900 ms, and then the warning stimulus was repeated. Individuals were instructed to give their response after the conclusion of the second warning stimulus.

It should be noted that the task design involved the 1600 ms period after the participants had heard the sentence and before they were required to respond, in order to avoid interference during the recording session. The onset of ERP recording was 500 ms after the end of the auditory presentation of the sentence (Table 1). According to previous studies, subjects performing the Hayling task required 2273 ± 542 ms for the initiation condition and 4760 ± 1450 ms for the inhibition condition [19], as well as 2570 ± 210 to 3180 ± 290 ms and 3400 ± 350 to 3720 ± 220 ms [28], respectively. Therefore, it is reasonable to consider that the participants of the study, at the target time window of 530 to 580 ms following the sentence presentation, were still in the process of combining storage and manipulation of the information, thus performing a working memory task [23,24].

**Table 1:** Sequence of events in each experimental trial.

Sequence of actions	Duration of actions
Auditory sentence presentation	3-5 sec
EEG recording	500 ms
Warning stimulus*	100 ms
ERP recording*^+^	1 sec
Warning stimulus repetition	100 ms
Response onset	Within 5 sec
Period between response completion and onset of next sentence presentation	4-9 sec

*Simultaneous onset of warning stimulus and of ERP recording

^+^Peak amplitudes were measured relatively to the mean amplitude of the 100 ms pre-stimulus baseline period; latency measurements were computed relatively to warning stimulus onset.

Each condition of the task contained 30 sentences. Before the ERP recording, there was a training period for each condition of the Hayling test in order for the participants to comprehend the nature of a correct response. This training period included the task instructions together with examples of correct responses followed by two training trials for both the initiation and the inhibition condition. If a participant had any difficulties in the understanding of the procedure, the phase of the training period was repeated. If a participant had difficulty in performing the task after the second training period he would be excluded from the particular ERP recording. All participants successfully passed the second training period. A trial for the response initiation condition was considered as incorrect if the word generated from the participant did not complete correctly the sentence. For the condition of response inhibition, a trial was considered as incorrect if the response plausibly completed the sentence or if the response had connection in some way to the sentence although it was not a direct completion of the sentence. A lack-of-response within 5 sec from the termination of the second warning stimulus was also considered as an incorrect trial. Guidelines for errors were based on the directions of Burgess and Shallice [18]. To avoid noise interference in ERP recording because of incorrect trials, participants who completed a total of 6 errors or lack of responses were disqualified. Thus, one individual from the initiation and four from the inhibition condition were excluded. From the remaining subjects, only the correct trials were included for further analyses. In the initiation condition the mean ± SD number of errors in the right- and the left-handed individuals who were included in the ERP analysis was 0.30 ± 0.60 (range, 0-2) and 0.36 ± 0.65 (range, 0-2), respectively (NS); in the inhibition condition it was 2.57 ± 1.45 (range, 0-5) and 2,38 ± 1.31 (range, 0-5), respectively.

### Experimental setup

A Faraday room was used in order to eliminate any electromagnetic interference that could effect the measurements; the attenuation of the mean field was more than 30 dB. EEG activity was recorded from 26 scalp Ag/AgCl electrodes based on the International 10-20 system of electroencephalography [29]. Linked ear lobes served as reference. Electrode resistance was kept constantly below 5 KOhm. The bandwidth of the amplifiers was between 0.05-35 Hz in order to avoid interference of the power supply network's signal, which is at 50 Hz. Eye movements were recorded with the use of electro-oculogram and recordings with EEG higher than 75 μV were excluded. The evoked biopotential signal was digitalized at a sampling rate of 1 KHz and was averaged by a computerized system. Signal recording, for each participant, lasted for a time period of 1500 ms at EEG leads Fp1, Fp2, Fpz, AFz, F7, F3, Fz, F4, F8, FC5, FC6, C3, C2, C1, T3, CP5, CP6, T4, P3, Pz, P4, T5, O1, Oz, O2, T6. The starting point of the recording was just after the conclusion of the auditory presentation of each sentence. The sequence of events was: sentence auditory presentation (duration 3-5 sec), EEG recording for 500 ms, onset of first warning stimulus (duration 100 ms) and parallel onset of ERP recording (duration 1000 ms), onset of second warning stimulus (duration 100 ms), oral response of participant after termination of second warning stimulus (for correct answer onset of response within 5 sec) (Table 1). For avoiding habituation with the conditions of the test, the onset of the next sentence auditory presentation varied between 4-9 sec from the completion of the previous oral response.

The ERP amplitudes for each electrode were averaged reference using as baseline the voltage over the 100 ms pre-stimulus epoch. An algorithm was used for identifying the positive peak between 30 and 80 ms after the onset of the first warning stimulus. Also, the Low Resolution Electromagnetic Tomography (LORETA) software, that provides three-dimensional images of brain electrical activity [30], was applied to study the activation density of the P50 component during the test in both right- and left-handers.

### Measure of handedness

In order to examine two well-differentiated groups, left-handers and right-handers, we used a two level procedure. The screening of the first level required three questions, hand writing, hand throwing, and hand used for holding knife without a fork. A left- or right-hand preference in all three questions was required for participation in the study and admission in the initial left- or right-handed group. In addition, for right-handers a positive answer in the question of strong right-handedness was required. Thus, most ambidextrous were excluded from the initial sample of participants.

The second level of the procedure for the assessment of handedness required the application of the EHI in the initial groups of left- and right-handers. The criterion applied for the left-handed group was an H< 0 [27]. For a participant to be considered as right-handed an H >50 was required. By using these criteria, two individuals were defined as ambidextrous and were excluded from the analyses. A smaller degree of left-handedness was chosen as the cut-off value for the left-handed group because of the less lateralized nature of sinistrals [1-3] and the possibility of some adaptation by left handed people to a world predominantly organized for the right-handers [27].

### Statistical analysis

The main dependent variables are the P50 amplitudes and latencies of the 26 electrodes of the two conditions (initiation condition/inhibition condition) of the Hayling test expressed in μV and ms, respectively. Normality was tested by the Kolmogorov-Smirnov method and was shown that the dependent variables did not deviate from normal distribution. For this reason, values appear as mean ± SD and parametric statistical tests were applied. Two multivariale analyses of variance (MANOVA) models were applied, one on the latencies and one on the amplitudes, for all 26 electrode sites together. Latencies and amplitudes were considered as dependent variables, with Hayling condition (initiation condition/inhibition condition), handedness (left/right), and their interaction as the independent factors. Post-hoc pairwise comparisons with Bonferroni correction were carried out in case of significant effect. In order to examine the effect of hemispheric lateralization, the amplitudes of the ten pairs of the counterpart left and right electrodes, considered as within-subjects factor, were subjected to a repeated measures general linear model (GLM) model, with handedness and Hayling condition as the independent between-subjects factors, followed by post-hoc pairwise comparisons with Bonferroni correction between the electrode pairs. Gender was also introduced in the analysis as an independent factor. Statistical Package for the Social Sciences (SPSS) version 15 (Chicago, IL) was used to analyze the data.

Also, for the analysis of the data of the two conditions of the Hayling test in right- and left-handers, the statistical mapping procedures [31] of the LORETA software were used. Voxel-wise comparisons were carried out between the groups of left- and right-handers in initiation and inhibition condition of the Hayling test, as well as between initiation and inhibition conditions in left- and right-handers in the post-stimulus period of 30-80 ms. Subsequently, for the time windows at which statistically significant differences were observed, the average LORETA images were compared voxel-wise in order to yield the locations at which significant differences in density were present. The solution space, based on the Talairach and Tournoux brain atlas [32], consisted of 2394 voxels with a spatial resolution of 7 mm.

Statistical significance for the SPSS and the LORETA analysis was set at the 0.05 level.

## Results

The mean ± SD age of the left- and right-handed individuals studied was 25.3 ± 4.1 (range, 20 to 34) years and 26.4 ± 3.1 (range, 22 to 34) years, respectively (NS). The mean degree of laterality in the 33 left-handers was -71.4 ± 27.7 (-10 to -100), and in the 31 right-handers it was 93.9 ± 12.8 (50 to 100).

**Figure 1:**
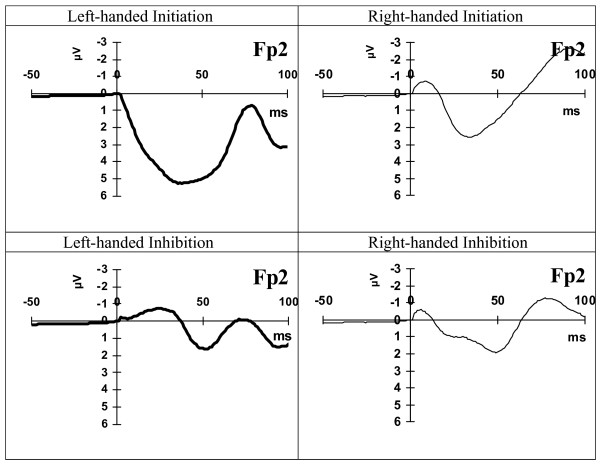
**Electrode Fp2 waveform**. Waveform of electrode Fp2 (μV) at the time window -50 - 100 ms from a right- and left-handed subject during the initiation and the inhibition condition.

Figure 1 illustrates the waveform of the representative electrode Fp2 at the time window -50 - 100 ms from a right- and a left-handed subject during the initiation and the inhibition condition.

The MANOVA model applied with the latencies of the 26 electrodes as dependent variables and handedness, Hayling condition, and their interaction as independent factors showed absence of a significant effect of the independent factors on the P50 time latencies.

The MANOVA model applied, with the amplitudes of the 26 electrodes as dependent variables, and handedness (right/left), Hayling condition (initiation condition/inhibition condition), and their interaction as independent factors showed a significant effect of the interaction of Hayling condition and handedness on the amplitudes of the electrodes [F(26,93) = 1.711 p = 0.033]. Post-hoc pairwise comparisons with Bonferroni correction between initiation and inhibition condition of the Hayling test for the right- and the left-handers showed significant differences on 13 of the 26 electrodes for the left-handers, but no significant differences for the right-handers. Significant differences were observed on 10 frontal electrodes (Fz, AFz, F3, F4, Fp2, Fpz, Fp1, FC5, F7, FC6), 2 central electrodes (C4, C3) and 1 temporal electrode (T3). Also, post-hoc pairwise comparisons with Bonferroni correction between the right- and the left-handers in initiation and inhibition condition of the Hayling test showed significant differences on 19 of the 26 electrodes in initiation condition, but no significant differences between the electrodes in inhibition condition. Significant differences were found on 10 frontal (Fz, FC5, F3, F7, F8, F4, AFz, Fp2, Fpz, Fp1), 5 central (CP5, Cz, C4, CP6, C3), 2 temporal (T3, T5) and 2 parietal electrodes (P3, Pz).

Also, gender was introduced in the analysis as independent factor and it was found to have no main effect or interaction effect with the other independent factors on the dependent variables of the study.

**Figure 2:**
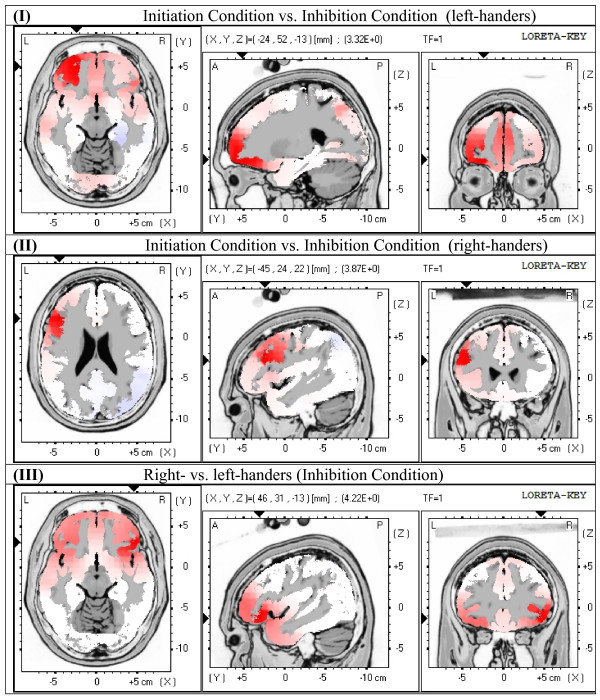
**LORETA images of voxel-wise comparisons**. LORETA images of voxel-wise comparisons between: (I) initiation and inhibition condition in the left-handers; (II) initiation and inhibition condition in the right-handers; and (III) right- and left-handers in inhibition condition, averaged within the time window 38-54 ms, 45-65 ms, 31-58 ms, respectively.

With the use of the LORETA voxel-by-voxel analysis, t-test comparisons were carried out between: (a) left-handers (initiation condition vs. inhibition condition, pairwise comparisons), (b) right-handers (initiation condition vs. inhibition condition, pairwise comparisons), (c) initiation condition (right-handers vs. left-handers, independent samples comparisons), and (d) inhibition condition (right-handers vs. left-handers, independent samples comparisons). Between initiation and inhibition condition in the left-handers, statistically significant differences were found at the time-window 38-54 ms (p = 0.0382). Maximum difference, with higher activation in initiation condition, was observed at X = -24, Y = 52, Z = -13, which corresponds to Broadmann area 11, left middle frontal gyrus, frontal lobe. Between initiation and inhibition condition in the right-handers, statistically significant differences were observed at the time-window 45-65 ms (p = 0.0044). Maximum difference, with higher activation in initiation condition, was observed at X = -45, Y = 24, Z = 22, which corresponds to Broadmann area 46, left middle frontal gyrus, frontal lobe. Between right- and left-handers in initiation condition there were no time-frames with statistically significant differences, but there was a trend for higher activation in the left-handed group. Between right- and left-handers in inhibition condition, statistically significant differences were found at the time-window 31-58 ms (p = 0.0074). Maximum difference, with higher activation in the right-handers, was observed at X = 46, Y = 31, Z = -13, which corresponds to Broadmann area 47, right inferior frontal gyrus, frontal lobe. The maximum difference between initiation and inhibition condition was greater in the right- than in the left-handers, with significant differences appearing in a restricted area of the left frontal lobe. On the other hand, left-handers exhibited a wider spread of significant differences between the two conditions of the test, which extended, though to a lower degree, and to the right hemisphere. Figure 2 illustrates the voxel-wise comparisons between initiation and inhibition condition in the left-handers (I), between initiation and inhibition condition in the right-handers (II), and between right- and left-handers in inhibition condition (III), averaged within the time window 38-54 ms, 45-65 ms, 31-58 ms, respectively.

The GLM repeated measures procedure showed that significant differences between left and right electrodes were observed only for the right-handers in the initiation condition (F(1,117) = 4.496, p = 0.036). Post-hoc comparisons showed that significant differences existed between the electrodes sites F7 and F8, F3 and F4, CP5 and CP6, P3 and P4 in the right-handed group during the initiation condition; higher amplitudes were recorded on electrodes F8, F4, CP6 and P4, all of them placed in the right hemisphere.

**Figure 3:**
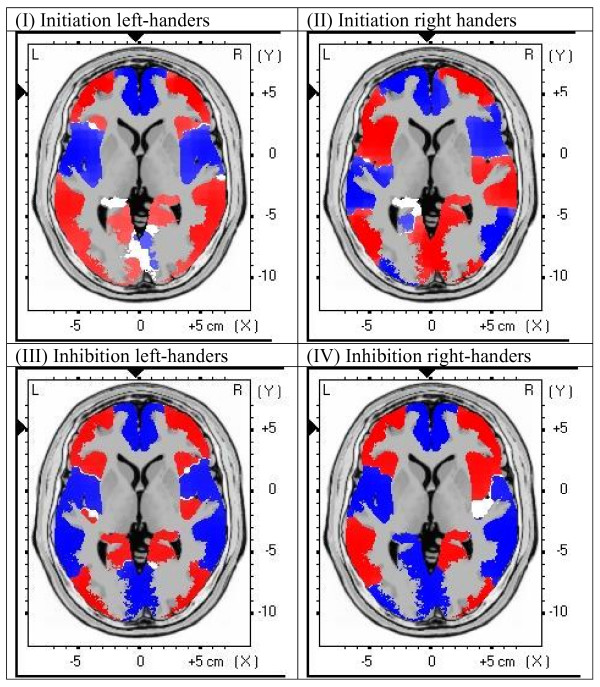
**LORETA images of mean activation**. LORETA images of mean activation in the P50 ERP window in: (I) initiation condition in left-handed group; (II) initiation condition in right-handed group; (III) inhibition condition in left-handed group; and (IV) inhibition condition in right-handed group.

Figure 3 illustrates the mean activation maps during the P50 ERP time-window, calculated with the LORETA software, in the initiation condition for the left-handed group (I), in the initiation condition for the right-handed group (II), in the inhibition condition for the left-handed group (III), and in the inhibition condition for the right-handed group (IV). Maximum activation was achieved at (X, Y, Z) = (-3, 52, 1), corresponding to Broadmann area 10, anterior cingulate, limbic lobe.

## Discussion

The findings of this study show greater P50 ERP amplitudes in the left- than in the right-handers in the initiation condition, with the most profound differences located at frontal leads, as well as reduced amplitudes in the inhibition than in the initiation condition, in the left-handed group. With LORETA analysis, the comparison between initiation and inhibition condition showed a reduced activation in the frontal lobes during the inhibition condition, with maximum differences focused in the left middle frontal gyrus, in both left- and right-handers. Moreover, LORETA revealed that in the inhibition condition right-handers had a significantly greater activation than left-handers in the frontal lobes, especially in the right inferior frontal gyrus. In left-handers, the reduced brain activation in the inhibition condition, when compared to the initiation condition, was observed by both the LORETA method and the P50 amplitudes. It is noteworthy that the electrode sites with the greatest reduction were located at frontal regions, and the LORETA method yielded a reduction of density activation in the frontal lobes as well. In reference to hemispheric lateralization, greater amplitudes were observed at the right-hemisphere in the right-handers, during the initiation condition. Also, illustration of the hemispheric activity by LORETA analysis during the P50 ERP time-window showed symmetrical activation patterns between the two hemispheres in both the initiation and the inhibition condition in left-handers, whereas in right-handers an asymmetrical activation of the hemispheres was observed. Regarding the differences between right- and left-handers in the patterns of reduction in the activity in the inhibition when compared to the initiation condition, LORETA analysis showed that the magnitude of the reduction was greater and more focused in right-handers, whereas left-handers presented significant differences, though weaker, that extended into wider areas of the frontal lobes.

The apparent discrepancies between the P50 amplitudes and the LORETA results may be explained by the fact that the two procedures investigate the P50 ERP component by a different approach, and provide complementary results. Thus, while LORETA depicts differences of cerebral activity manifested at the same time, taking into account in each time unit (1 ms) the voltages of all electrodes as a total, the P50 amplitudes indicate the maximum voltage recorded within the post-stimulus time period of 30-80 ms of each electrode.

The results of this report may be interpreted in the light of studies related to the Hayling test, the specifications of the modified version of the test applied in the current study, and the P50 ERP waveform. In reference to foregoing studies utilizing the Hayling test, positron emission tomography (PET) methodology showed a greater frontal activation during the initiation than the inhibition condition suggesting that the former might rely less on high levels of linguistic processing and more on low levels of word production, thus generating a functional pattern that could lead to higher frontal activation [33]. Collette et al. [19] using PET during the application of another version of the Hayling test found increased activity in prefrontal areas during the inhibition as compared to the initiation condition. The inconsistency between the findings of the two studies was attributed to differences in the modified forms of the Hayling test applied [19]. Hayling test is a measure of executive functioning, especially in relation to the WM operation [34] as applied in the current study, and although it has been suggested that prefrontal cortex possesses a pivotal role in executive control [35,36], research evidence emphasizes the importance of additional brain areas, such as broad cortical and subcortical networks, including thalamic pathways [37]. This broader view might result from the fact that the tests applied for assessing executive functioning are complex and induce a wide range of skills, thus complicating efforts to identify a unitary interpretation framework [37].

It is interesting to note that thalamic circuits link peripheral sensory systems to the cerebral cortex through 'feed-forward' relay neurons, while the major source of excitatory synapses in the thalamus is not afferent synapses from the periphery, but from the cerebral cortex itself [38-40]. An additional corroboration to this idea is provided by the synchronous adaptive resonance theory (SMART) [41]. According to this theory, the match between bottom-up adaptively filtered input patterns and learned top-down expectations causes gamma oscillations, whereas a mismatch between bottom-up and top-down signal patterns prevents the development of such a synchronous state. The P50 ERP component is thought to be part of the gamma band EEG synchronized response [8,11], and the initiation condition is an overlearned response for the sample studied. Hence, it follows that in the case of the initiation condition there is a matching between bottom-up filtered input patterns and top-down expectations that leads to the observed increase in P50 activation. This carries the possibility that in the inhibition condition the mismatch with the top-down expectations inhibits the development of such a synchronous state of gamma oscillations, a phenomenon that is mirrored by the observed reduction in P50 activation in the inhibition Hayling condition.

Further evidence indicates that thalamocortical networks are involved in the linguistic processing of syntactic and semantic aspects [20], in the regulation of higher-level lexical-semantic processes [42], as well as in the processing of high-level linguistic features, such as ambiguity resolution [21]. In conjunction with the foregoing studies, the reported pivotal involvement of the thalamus in executive control operations, as shown by discrete expression of thalamic ERP between go and no-go trials [43], combined with the reported alterations in thalamic activity in reference to handedness [3], may explain both the differences between initiation and inhibition condition in both right- and left-handers as well as the observed different patterns between right- and left-handers.

The different patterns of activity, as mirrored in this study by the P50 ERP as well as by the LORETA images between right- and left-handers in the initiation and the inhibition condition, could also reflect functional differences in regard to the hemispheric related associations of handedness [1-3]. In accordance with this view, Hund-Georgiadis et al. [1] using functional MRI showed a preponderance of the left inferior and middle frontal gyrus in right-handers, and bilateral activation patterns in left-handers, during the performance of language activation tasks. Similarly, Szaflarski et al. [2] reported a more symmetrical pattern of activity in the frontal lobes of left-handers. In a very recent study of our team, by applying the Stroop Color World task, it was observed that there are alterations in executive control in reference to handedness, thus supporting the view of a different brain functional organization between right- and left-handers [44].

Possible limitations of the present study lie in whether these findings represent state rather than trait effects, which appears to be a reasonable target for future research. Also, the participants were exclusively highly educated young adults and belonged to two well distinct handed groups, with ambidextrous individuals excluded. Under these terms, the sample studied is not indicative of the whole spectrum of handedness, but conversely the participants belonged to well-defined subpopulations and, thus, any group differences could be linked to alterations in brain organization related to handedness. However, future research should replicate the main findings in independent samples as well as further explore whether the findings are associated in a task-specific manner or across tasks. Finally, forthcoming studies controlling for age, trait, and state parameters, in conjunction with additional experiments that combine the time resolution of ERP with the spatial resolution of brain imaging techniques, may lead to clearer definitions of brain functions extending the findings of this study.

## Conclusion

An advantage of this work, that renders originality, is that brain activation in the time-window of P50 ERP was investigated by the application of a modified version of the Hayling test adjusted to induce working memory operation. To our knowledge this is the first investigation of P50 ERP in the process of a linguistic related test measuring executive control. The findings of this study lend support to the notion that thalamocortical function, as reflected by P50 ERP waveforms elicited during WM operation involving initiation and inhibition processes, appears to be related to handedness.

## Abbreviations

ERP: event-related potential; WM: working memory; LORETA: low resolution electromagnetic tomography; GBR: gamma-band response; EHI: Edinburgh handedness inventory; EEG: electroencephalogram; MANOVA: multivariate analyses of variance; GLM: general linear model; SPSS: statistical package for the social sciences; PET: positron emission tomography; SMART: synchronous adaptive resonance theory.

## Competing interests

The authors declare that they have no competing interests.

## Authors' contributions

INB, AR, and CP designed the study. INB and CP executed the study. EDN, CH, AEM, CNK and CP programmed and established the experimental procedure. INB, EDN, CH, AEM and CNK, processed and analyzed the data. INB and CP interpreted the results and drafted the manuscript. AR and GNP coordinated the study and critically revised the manuscript. All authors were involved in the editing and approval of the final manuscript.
